# Buckling Performance Evaluation of Double-Double Laminates with Cutouts Using Artificial Neural Network and Genetic Algorithm

**DOI:** 10.3390/ma17194677

**Published:** 2024-09-24

**Authors:** Ruiqing Ju, Kai Zhao, Carol A. Featherston, Xiaoyang Liu

**Affiliations:** 1 School of Civil Engineering, Qingdao University of Technology, Qingdao 266033, China; 2 School of Engineering and Architecture, University College Cork, T12 HW58 Cork, Ireland; 3 School of Engineering, Cardiff University, Queen’s Buildings, The Parade, Cardiff CF24 3AA, UK

**Keywords:** double-double laminates, artificial neural network, genetic algorithm, optimisation, cutouts

## Abstract

Although the double-double (DD) laminates proposed by Tsai provide a promising option for achieving better structural performance with lower manufacturing and maintenance costs, the buckling performance of perforated DD laminates still remains clear. In this study, optimal ply angles, rotation angles, and the corresponding maximum buckling loads are determined for DD laminates with various cutouts, which are used for comparisons to evaluate the effects of cutout size and shape on the buckling behaviour of perforated DD laminates. Apart from conventional circular and elliptical cutouts, the use of a combined-shape cutout for DD laminates is also investigated. As a large number of optimisations are required to obtain the maximum buckling loads for different cases in this study, an efficient optimisation method for perforated DD laminates is proposed based on an artificial neural network (ANN) and a genetic algorithm (GA). Unlike conventional quadaxial (QUAD) laminates, the repetition of a four-ply sublaminate in DD laminates makes their layup to be represented by only two ply angles; hence, the application of ANN models for predicting the buckling behaviour of various perforated DD laminates is studied in this paper. The superior performance of the ANN models is demonstrated by comparisons with other machine learning models. Instead of using the time-consuming FEA, the developed ANN model is utilised within a GA to obtain the maximum buckling load of perforated DD laminates. Compared to the circular cutout, the use of elliptical and combined-shape cutouts leads to more noticeable changes in the optimal ply angles as the cutout size increases. Based on the obtained results, the use of the combined-shape cutout is recommended for DD laminates.

## 1. Introduction

Composite laminates are used extensively in several engineering domains, including aerospace, automotive, marine, and civil. In practical engineering, strategically positioning cutouts in these laminates is essential for ventilation, maintenance, and installation of cables and pipes [1,2,3]. However, the presence of cutouts inevitably reduces buckling resistance, which is a great concern in the design of composite laminates [4,5].

Extensive research has been conducted on the structural behaviour of composite laminates with cutouts, mainly focusing on strength [6,7,8,9] and buckling behaviour [10,11,12,13,14,15,16]. Nemeth [5] conducted a preliminary investigation on the buckling behaviour of rectangular laminates with a centrally located circular cutout, based on which an approximate method for predicting the buckling load of perforated laminates was developed. Silveira et al. [17] investigated the buckling resistance of laminates with elliptical cutouts using the constructal design method. Shojaee et al. [18] employed an isogeometric finite element method for the buckling analysis of laminates with a cutout of complicated shape. In the work of Taheri–Behrooz et al. [19], experimental and numerical procedures were performed to investigate the effect of cutout size on the buckling behaviour of composite cylinders. In order to minimise computational cost, Tian et al. [20] conducted a buckling optimisation of laminates with multiple cutouts based on a surrogate-based stacking sequence optimisation framework. Hu and Lin [21] employed a sequential linear programming method to maximise the buckling load of laminates with cutouts, with the ply angles treated as design variables. In the work of Zhu et al. [22], the fibre path of composite laminates with cutouts was optimised to enhance the uniaxial tensile strength based on a trust-region-reflective algorithm. Apart from the studies discussed above, the effect of cutouts on the maximum displacement [23], stress distribution [24], and vibration behaviour [25,26,27,28] of laminates has also been investigated.

Since the structural performance of composite laminates is significantly affected by their layups, several promising layups have been proposed, such as the variable angle tow (VAT) [26,27,29,30] and double-double [31] layups. Numerous studies [32,33,34,35,36] have been performed on the analysis and optimisation of VAT laminates with cutouts. Niu et al. [37] experimentally investigated the tensile properties of VAT laminates with circular cutouts. In the work of Milazzo et al. [38], a single-domain Ritz formulation was developed to investigate the buckling and post-buckling behaviour of VAT laminates with cutouts. Passos et al. [39] conducted a buckling load optimisation of stiffened VAT laminates with cutouts with a surrogate model based efficient global optimisation (EGO) algorithm developed to reduce the computational cost. In order to determine the effect of cutouts on the strength and buckling performance of VAT laminates, Lopes et al. [40] conducted a comprehensive numerical analysis based on finite element analysis (FEA). Although FEA is commonly recognised as the most powerful and versatile analysis method, it incurs a substantial computing expense, particularly when being used in iterative optimisation processes that necessitate a considerable number of analyses. Recently, the application of artificial neural network (ANN) models to the performance evaluation [41,42,43,44,45,46] and optimised design [47,48] of composite laminates has been attracting increasing attention from researchers. Instead of using FEA for the structural analysis in the optimisation, the application of the ANN models trained based on the FEA results significantly improves the optimisation efficiency [49,50,51,52,53,54,55,56,57,58,59,60], providing a potential solution for the optimisation of composite laminates with cutouts of complicated shapes.

**Figure 1:**
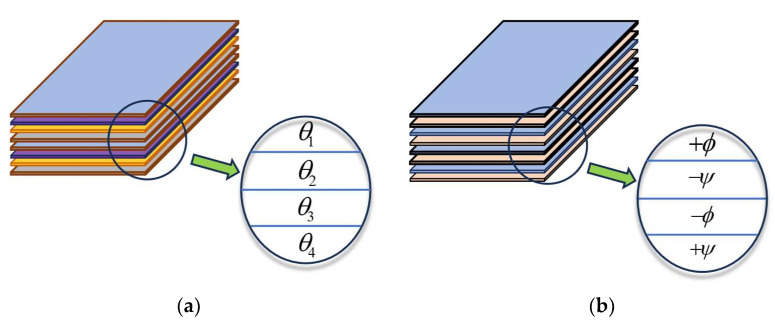
The layups of (**a**) QUAD laminates and (**b**) double-double laminates.

In order to achieve better mechanical performance with lower manufacturing and maintenance costs, a novel composite laminate referred to as a double-double (DD) laminate was proposed by Tsai [31]. Unlike conventional quadaxial (QUAD) laminates, which consist of a set of plies with predefined angles usually limited to 0°, 90°, +45°, and −45°, as shown in Figure 1, DD laminates comprise a repeat of a four-ply sublaminate, of which the layups could be one of the following four types: $\left[+\phi /-\psi /-\phi /+\psi \right]$, $\left[+\phi /+\psi /-\phi /-\psi \right]$,$\left[+\phi /-\psi /+\psi /-\phi \right]$ and $\left[\pm \phi /\pm \psi \right]$, in which ϕ and ψ represent two ply angles with continuous values ranging from 0° to 90°. DD laminates have been proven to be a promising substitute for QUAD laminates, as they can easily achieve homogenisation, tapering, and efficient optimisation [45,59,60,61,62]. Several studies have been performed to investigate the mechanical performance of DD laminates in terms of buckling [63], postbuckling [64], crashworthiness [65], and low velocity impact behaviour [66]. Although the presence of cutouts is inevitable in DD laminates in practical engineering, the effect of cutouts on the buckling performance of DD laminates has not been investigated. Therefore, in this study, the buckling behaviour of DD laminates with different cutouts (i.e., circular, elliptical, and a combined shape consisting of a rectangle in the middle with two semiellipses at both ends) is investigated. In addition, the application of machine learning models to predict the buckling load of DD laminates with two ply angles and cutout dimensions as inputs is studied. The ANN model with high prediction accuracy is then employed within GA to obtain the optimal layup and cutout shapes to maximise the buckling load. Based on the obtained results, the buckling performance of different types of perforated DD laminates is investigated. Section 2 of this paper introduces the proposed prediction and optimisation procedures. The obtained results are outlined and discussed in Section 3, and conclusions are provided in Section 4.

## 2. Methodology

**Figure 2:**
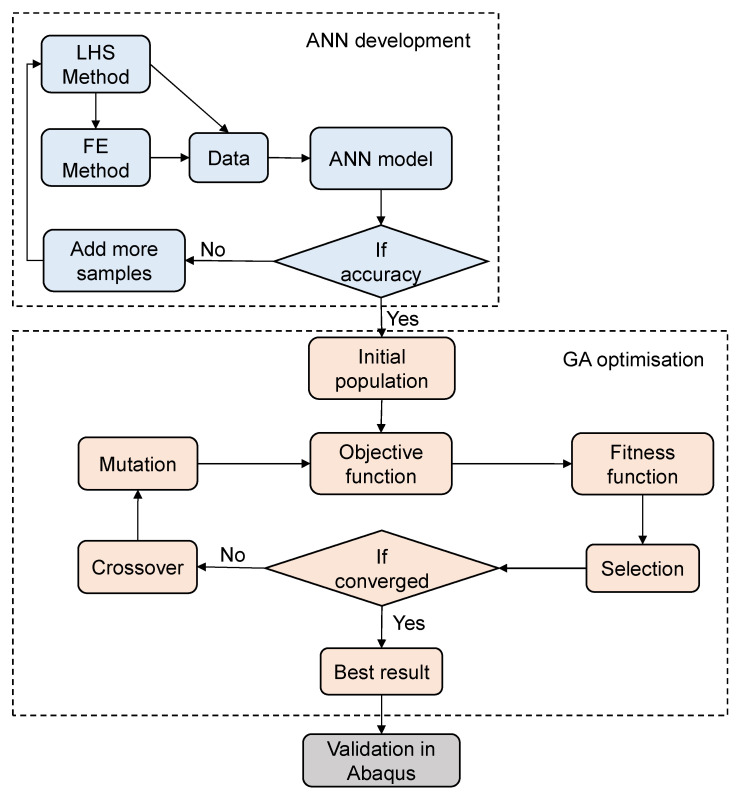
Framework of the proposed method.

In this section, the method proposed for obtaining the optimal layup and cutout configuration of DD laminates is introduced. First, the Latin hypercube sampling (LHS) method and FEA are employed to develop a database for training and testing the ANN models. The input combinations of the ANN model (i.e., layups and cutout configurations) are determined using the LHS method, while the corresponding outputs (i.e., buckling load) are obtained using the FEA. Based on the developed database, ANN models that are capable of predicting the buckling load of DD laminates with various cutouts are developed. Subsequently, these ANN models are employed to obtain the maximum buckling load of the perforated DD laminates during the GA optimisation. Figure 2 presents the framework of the proposed method.

### 2.1. Database Generation

**Figure 3:**
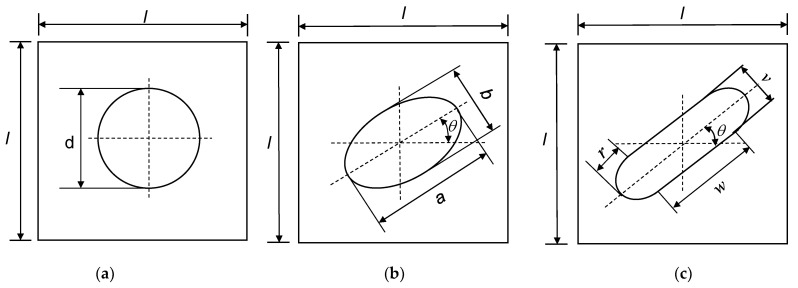
Configurations of three types of cutouts: (**a**) circular cutout; (**b**) elliptical cutout; (**c**) combined-shape cutout.

In this paper, one of the four types of DD laminates, commonly referred to as *staggered 1* with the stacking sequence of $\left[+\phi /-\psi /-\phi /+\psi \right]$ is applied. As shown in Figure 3, three types of cutouts at the centre of a square DD laminate are considered. Apart from the layup, the cutout diameter d is also set as a design variable for the laminate with the circular cutout. For the laminate with the elliptical cutout, the longest diameter a and shortest diameter b along the major and minor axes, respectively, are both considered. The ellipse is also allowed to rotate around the centre; hence, the rotation angle θ is also set as a design variable with a range between 1° and 180°, as shown in Figure 3b. Figure 3c depicts the laminate with the combined-shape cutout, for which the length w and width v of the rectangle, the radius of the semi-ellipse along the major axis r, as well as the rotation angle θ are set as design variables. The rotation angle θ for the elliptical and combined-shape cutouts are the same.

Latin hypercube sampling (LHS) is a statistical method used to generate a sample of inputs from a multidimensional distribution. The range of each variable is divided into equal intervals, and samples are drawn such that each interval is sampled exactly once [67]. The design variables described above are taken as the inputs for the machine learning models, the value combination of which is determined using the LHS approach to ensure a uniform distribution of data points. The buckling load is determined by implementing a buckling analysis using the finite element software ABAQUS 2022 [68], and a Python script is employed to conduct the large number of FEA. The data samples in the database are normalised to ease the training process. Furthermore, the database is partitioned into two sets; 80% of the data samples are assigned to the training dataset, while the remaining 20% are assigned to the testing dataset.

### 2.2. Artificial Neural Networks

**Figure 4:**
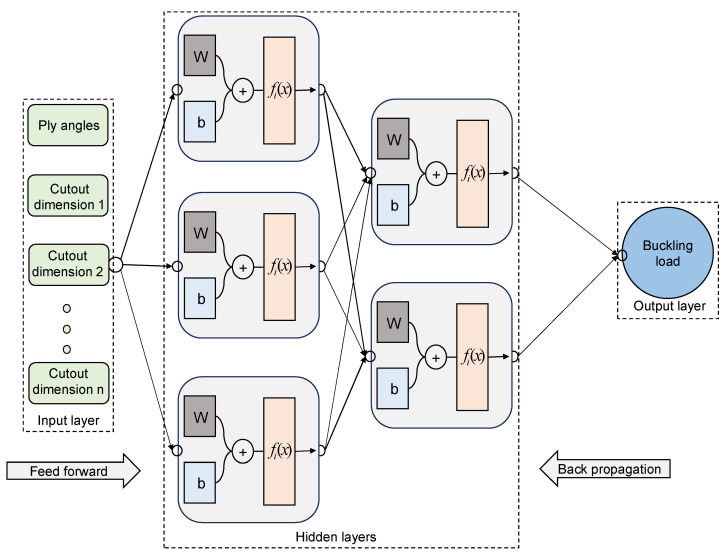
The structure of ANN model.

Derived from the structural framework of the biological neuron system, ANNs emerge as a formidable technique for solving highly non-linear relations with high accuracy and efficiency. As shown in Figure 4, an ANN comprises an input layer, an output layer, and several intervening hidden layers, each possessing a specific quantity of neurons, with these neurons connected to both the preceding and subsequent layers. The creation of an ANN model involves a training procedure wherein the neural networks learn to discern the intricate connections between inputs and outputs. 

In this paper, the model development is facilitated by Keras [69], an open-source Python package. Since the inputs are the ply angles ϕ and ψ, and also the dimension parameters of the cutouts, the quantity of neurons in the input layer is determined by the type of the cutout, whereas the output layer contains only one neuron representing the buckling load $N_{b}$. The parameters of the ANN models, including the number of hidden layers and the corresponding number of neurons, are set using a trial-and-error approach. Once the inputs are received, they flow from the input layers to the output layers, undergoing multiplication by weight and the incorporation of bias at each neuron. The output of each neuron, which is determined based on an activation function $f_{i}$ (e.g., linear function, sigmoid function, ReLU function etc.), can be expressed as

(1) $$Output=f_{i}(\sum_{j=1}^{n}w_{ij}p_{j}+b_{i})$$

where $w_{ij}$ and $b_{i}$ represent the weight and bias for the *i*th neuron in the current layer, $p_{j}$ is the input from the *j*th neuron from the preceding layer, and *n* is the number of neurons in the preceding layer.

In the present study, a linear activation function that produces an output value identical to its input is employed for the output layer. The input layer utilises the softplus activation function, which is expressed by Equation (2), while the *ReLU* function, as shown in Equation (3), is utilised for the intervening hidden layers.

(2) $$softplus(x)=\log(\sqrt{x}+1)$$

(3) $$ReLU(x)=\left\{\begin{array}{cc} x & \left(x>0\right)\\ 0 & \left(x\leq 0\right) \end{array}\right.$$

where x represents the input of the activation function. Following the generation of the output at the final layer, the discrepancy between the achieved output and the goal output is minimised by optimising the weights and biases using a backpropagation procedure. The loss function used in this paper is expressed in Equation (4), which is minimised during the training process using the Adam optimiser [70].

(4) $$loss=\frac{1}{n}\sum_{i=1}^{n}{\left(t_{i}-p_{i}\right)}^{2}$$

where *n* represents the number of data samples in the training dataset. $t_{i}$ is the target for the *i*th sample, and $p_{i}$ is the corresponding result obtained by the ANN model.

**Figure 5:**
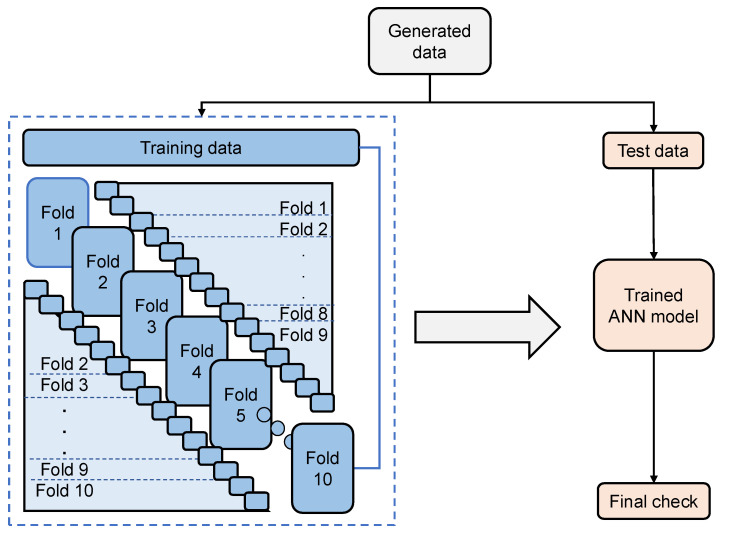
The structure of the 10-fold cross-validation.

In this study, a tenfold cross-validation procedure is implemented to improve the performance of the ANN models. The training dataset is divided into ten subsets, based on which the models are trained ten times with the data samples in nine subsets, and the remaining dataset is used for validation. The ten datasets are sequentially used for validation, as shown in Figure 5.

The performance of the trained ANN models is assessed with regard to the coefficient of determination (*R*^2^) and the root mean square error (*RMSE*), the expressions of which are shown in Equations (5) and (6), respectively.

(5) $$R^{2}=1-\frac{\sum_{m=1}^{n_{t}}{\left(t_{m}-p_{m}\right)}^{2}}{\sum_{k=1}^{n_{t}}{\left(t_{k}-\bar{t}\right)}^{2}}$$

(6) $$RMSE=\sqrt{\frac{1}{n_{t}}\sum_{i=1}^{n_{t}}{\left(t_{i}-p_{i}\right)}^{2}}$$

where $n_{t}$ represents the number of testing data samples and $\bar{t}$ represents the average value of the target values.

### 2.3. Comparative Machine Learning Models

The application of two other commonly used machine learning models, linear regression (LR) and random forest (RF), is also investigated. LR provides prediction results based on a linear combination of the input features, with variations in the output assumed to be proportional to variations in the inputs. Its ease of development contributes to its widespread application in prediction problems. RF is developed by constructing a set of decision trees, which involves representing attributes with internal nodes, the branches of which denote the values of the attribute tested. The prediction of the RF is determined by aggregating the predictions of the individual trees. A trial-and-error approach is again adopted to determine the parameters of the two models. The inputs, outputs, and dataset for training and testing of these two models are the same as those for the ANN models.

### 2.4. Optimisation Procedure

The aim of the optimisation is to obtain the maximum buckling resistance of DD laminates with various cutouts. The optimisation problem is expressed below:

(7) $$\begin{array}{l} \mathrm{find}\;:x\\ \mathrm{maximise}:N_{b}(x)\\ x_{i}^{l}\leq x_{i}\leq x_{i}^{u}(i=1,2,3,\ldots ,n_{d}) \end{array}$$

where $N_{b}(x)$ is the critical buckling load, *x* represents the design variable vector, which includes the design variables introduced in Section 2.1, $n_{d}$ represents the total number of design variables; and $x_{i}^{l}$ and $x_{i}^{u}$ represent the lower and upper limits of each design variable, respectively.

As a heuristic optimisation technique, GA inspired by biological reproduction is employed in this study. Instead of using the gradient information of Equation (7), a stochastic search is performed based on a population of designs. Initially, a population of random designs is generated. After that, the proposed ANN model is used to evaluate the buckling resistance of each individual. Since the aim of the optimisation is to maximise the buckling load, individuals with a higher value of buckling load are assigned with a higher fitness value. Following this, a roulette wheel method incorporated within an elitism procedure is applied to the selection process, during which individuals with a higher fitness value have a greater probability of being selected into the next generation. In order to generate a new generation, a two-point crossover is carried out on the selected individuals, after which a mutation operation is performed. This process repeats until the stopping criterion is met. A trial-and-error approach is adopted to determine the GA parameters, including the probabilities of crossover and mutation, number of elites, population size, etc. Figure 2 shows the integration of the GA optimisation.

## 3. Results and Discussions

**Figure 6:**
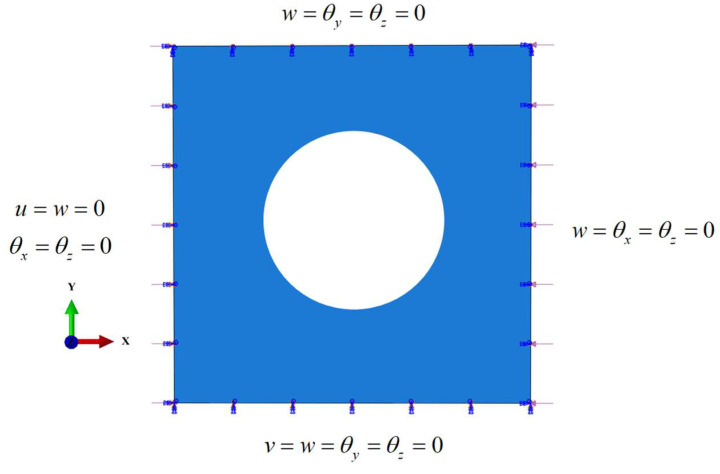
Loading and boundary conditions.

Here, the method proposed in the last section is applied to DD laminates with three types of cutouts. Table 1 presents the ranges of the design variables, with dimensions varying in increments of 10 mm, and angles varying in increments of 1°. The dimensions of the square plates remain constant at a length of 100 mm. The laminates are assumed to have 16 plies in total, and the thickness of each is set to be 0.125 mm. To generate the database, ABAQUS is employed to obtain the buckling loads of the DD laminates, using the tri S4R shell element with six degrees of freedom at each node. In addition, a mesh sensitivity analysis is implemented herein, based on which the mesh size is set to be 1 mm. The material properties selected from [21] are outlined in Table 2. Figure 6 shows the loading and boundary conditions.

### 3.1. Validation of FEA

**Figure 7:**
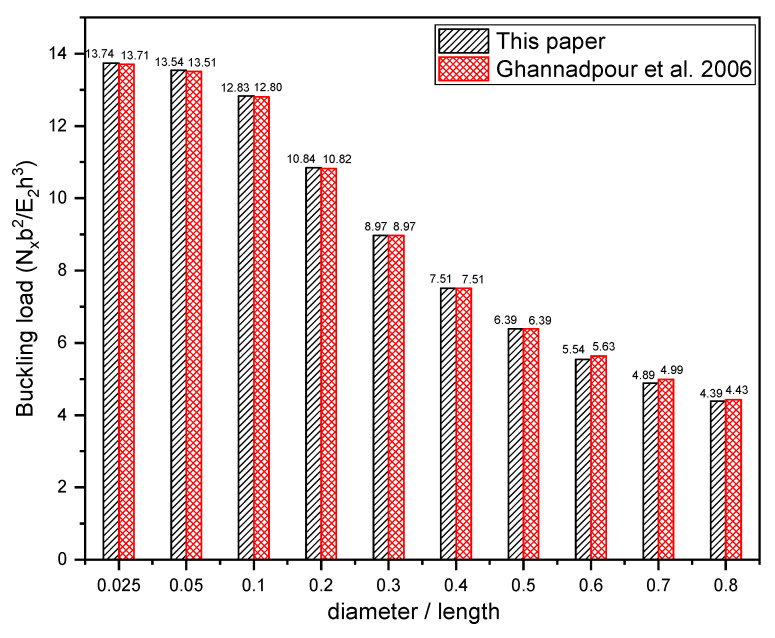
Validation of the FEA through the results presented in [24].

Prior to the development of the database, the FEA used in this paper is verified by the comparison with the results provided in Ref. [24]. A total of ten perforated square laminates with circular cutouts of varying sizes are employed for comparison. The dimension of the laminate is 120 mm × 120 mm, and the ratio of cutout diameter to laminate width varies from 0.025 to 0.8. The layup of the laminate is [0/90]_2s_, and the thickness of each ply is 0.15 mm. In-plane compressive load is applied along one direction of the laminate. The material properties and boundary conditions are set the same as those presented in Ref. [24]. Figure 7 shows the comparison of the buckling loads obtained in this paper with those provided in Ref. [24]. It can be easily observed that the results obtained in this paper are close to the results presented in Ref. [24], and the maximum difference between them for various cutout sizes is within 2%, illustrating the effectiveness of the FEA used in this paper.

### 3.2. DD Laminates with a Circular Cutout

**Figure 8:**
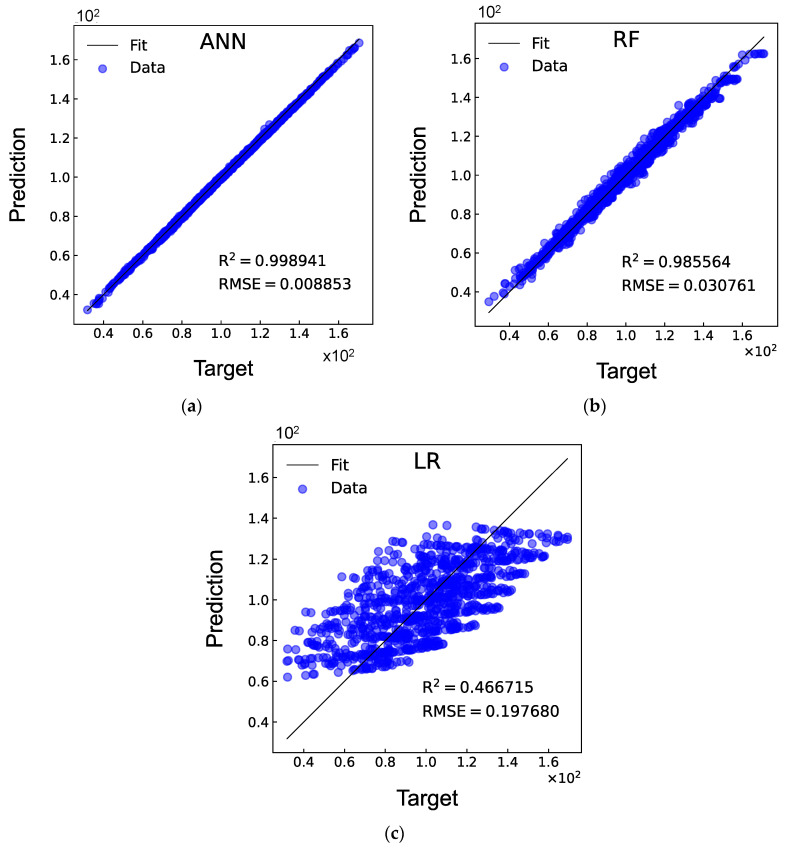
Comparisons of R^2^ and RMSE between the (**a**) ANN, (**b**) RF, and (**c**) LR models for DD laminates with a circular cutout.

The two-ply angles ϕ and ψ, and the cutout diameter d are taken as the design variables for the DD laminates with a circular cutout; hence, they serve as inputs for the machine learning models. The database contains 5000 data samples. Therefore, 5000 FEA are performed, with a computational time of approximately 55.03 h. Following a process of trial-and-error, the architecture of the ANN model is established as 3-30-30-30-30-30-1 (5 hidden layers, each containing 30 neurons). Table 3 shows the 10-fold cross-validation results of the ANN models. It can be observed that the values of R^2^ for both training and validation obtained in the 10 rounds are all close to 1, and the corresponding values of RMSE are around 0.009, indicating that the developed model performs consistently well across different folds of the database. The values of R^2^ and RMSE for the test results are consistent with the k-fold results. Figure 8 also presents comparisons between the ANN, RF, and LR models. While the RF model achieves prediction results with R^2^ and RMSE values of 0.986 and 0.031, respectively, it is notable that the ANN model outperforms it, exhibiting a higher R^2^ value and a lower RMSE value. As expected, the LR model exhibits the lowest R^2^ value and the highest RMSE value, demonstrating that the prediction of buckling load for DD laminates with a circular cutout is a highly non-linear problem that cannot be solved through a linear combination of the two-ply angles and cutout diameter. The obtained results indicate that in contrast to this, the developed ANN model is capable of predicting the buckling load with high accuracy.

**Figure 9:**
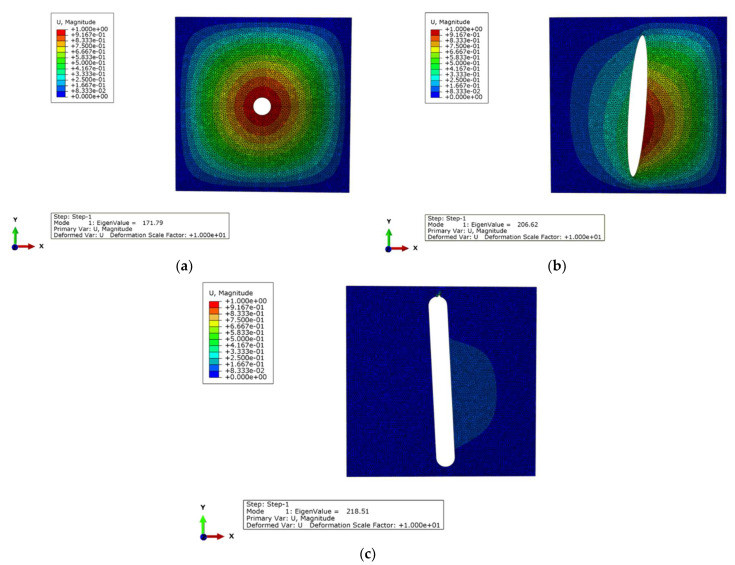
FEA results of the optimal DD laminates: (**a**) buckling mode of a DD laminate with a circular cutout; (**b**) buckling mode of a DD laminate with a elliptical cutout; (**c**) buckling mode of a DD laminate with a combined-shape cutout.

**Figure 10:**
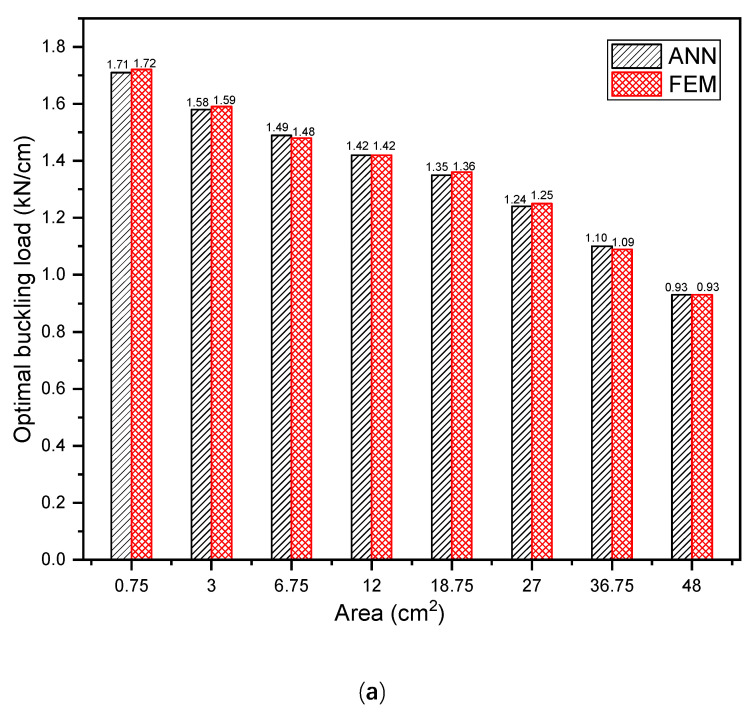

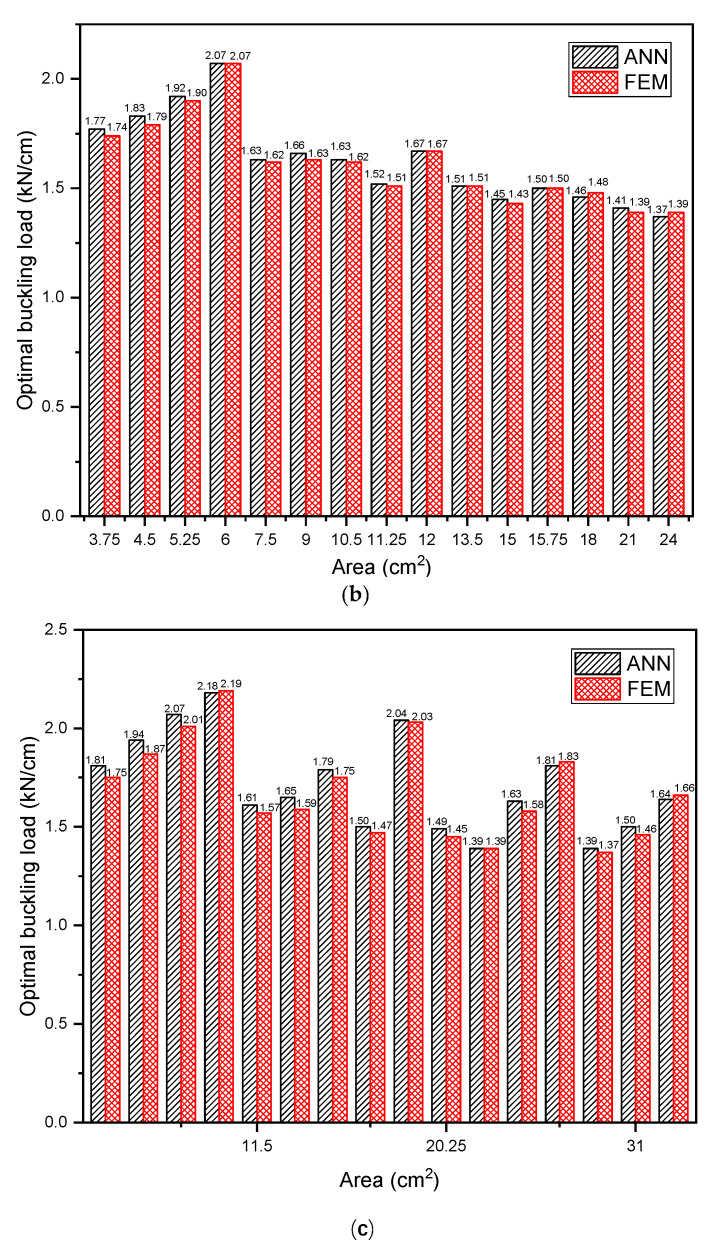
Maximum buckling loads of DD laminates with a cutout of varying area: (**a**) circular cutout; (**b**) elliptical cutout; (**c**) combined-shape cutout.

Since the GA performs optimisation based on a stochastic search, GA optimisation is run 10 times to ensure the final result is well converged. In this case, a population size of 50 individuals is applied, with the probability of crossover set at 0.8 and the probability of mutation set at 0.05. The maximum buckling load converges at 1.714 kN/cm, the optimal ply angles ϕ and ψ are 42° and 43°, respectively, and the corresponding cutout diameter d is 10 mm. Subsequently, ABAQUS is employed to conduct a buckling analysis on the optimised DD laminate. As can be seen from Figure 9a, the buckling load of the optimised DD laminate obtained by FEA is 1.718 kN/cm, further demonstrating the efficacy of the ANN model in buckling load prediction. Since the buckling optimisation tends to provide a cutout with the minimum size, the optimal ply angles for the perforated laminates with predefined cutout sizes are also obtained by fixing the cutout size during the optimisation. The optimal ply angles ϕ and ψ for different cutout sizes are presented in Table 4. It can be seen that the optimal values of ϕ and ψ vary slightly with the variation of the cutout size, but they are all around 40° for this study. Figure 10a shows the maximum buckling loads of the laminates with circular cutouts of different sizes. It can be seen that the buckling resistance of the laminates decreases significantly with the increase in the cutout area. In addition to this, it can be observed that the discrepancy between the results obtained by the ANN model and FEA remains negligible with the increasing cutout area.

### 3.3. DD Laminate with an Elliptical Cutout

**Figure 11:**
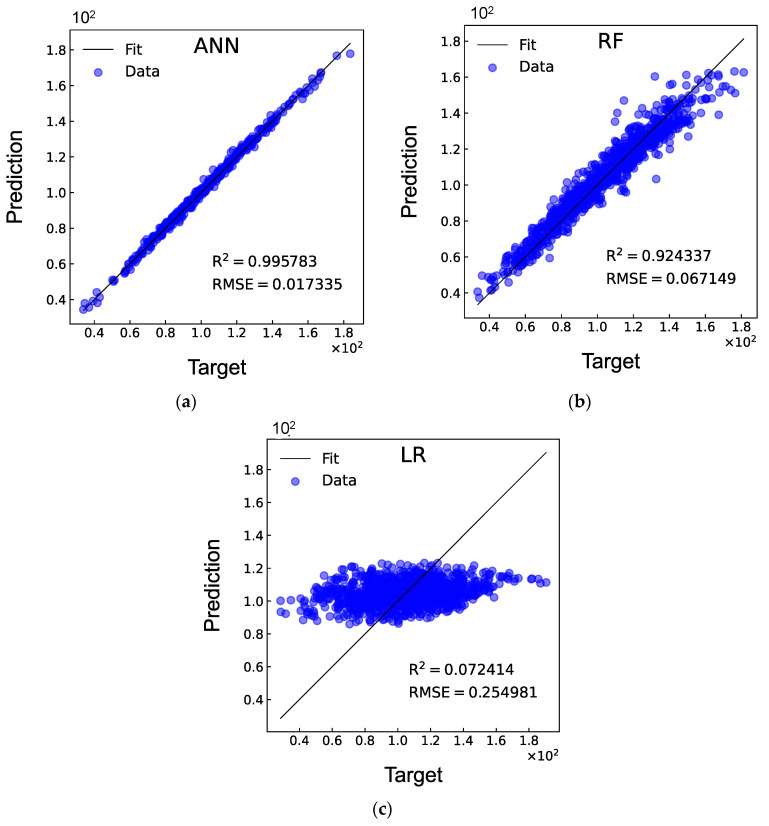
Comparisons of R^2^ and RMSE between the (**a**) ANN, (**b**) RF, and (**c**) LR models for DD laminates with an elliptical cutout.

The optimisation of DD laminates with an elliptical cutout, where the two-ply angles ϕ and ψ, the longest diameter a, shortest diameter b, as well as the rotation angle θ, are set as design variables, is explored next. To generate the database, 5000 FEA are performed with an overall computational time of approximately 58.97 h. The architecture of the ANN model, established through a trial-and-error strategy, is 5-50-50-50-50-50-1 (5 hidden layers, each containing 50 neurons). It can be observed from Table 3 that the values of R^2^ obtained in the 10 rounds are consistently close to 1, and the corresponding values of RMSE are around 0.017. The R^2^ and RMSE values obtained for the three models for the test database are shown in Figure 11. It can be observed that for the elliptical cutout, the ANN model achieves slightly higher RMSE values compared to the circular cutout, as more input features are taken into account. Comparing the three models indicates that the ANN model outperforms the others, with the advantage of using ANN models being more pronounced for the elliptical cutout than for the circular one.

The GA optimisation is implemented with a population size of 100 individuals, with the probabilities of crossover and mutation defined as 0.8 and 0.1, respectively. The maximum buckling load achieved in this section is 2.068 kN/cm, for which the ply angles ϕ and ψ are 37° and 84°, respectively, the rotation angle θ is 86°, and the dimensions a and b are 10 mm and 80 mm, respectively. As can be seen from Figure 9b, the buckling load of the optimised DD laminate obtained by FEA is 2.066 kN/cm. The optimal ply angles ϕ and ψ, and the corresponding rotation angle θ for several predefined cutout sizes are also obtained by fixing the dimension parameters during the optimisation process. The size of the elliptical cutout is determined based on the dimensions a and b; hence, all possible combinations of a and b within the ranges shown in Table 1 are selected herein. The optimal values of ϕ, ψ, and θ are outlined in Table 4. Unlike the circular cutout, the application of elliptical cutouts leads to relatively noticeable changes in the optimal ply angles as the cutout size increases. In addition, it can be observed that the optimal value of θ generally exhibits an ascending trend as the cutout size increases, suggesting the need for a greater rotation angle for a larger-sized elliptical cutout. Figure 10b presents the maximum buckling loads of these laminates with various cutout sizes. It should be noted that fluctuations in the maximum buckling loads occur with the increase of the cutout area, and this is because the cutout dimensions are predefined in the optimisation.

### 3.4. DD Laminate with a Combined-Shape Cutout

**Figure 12:**
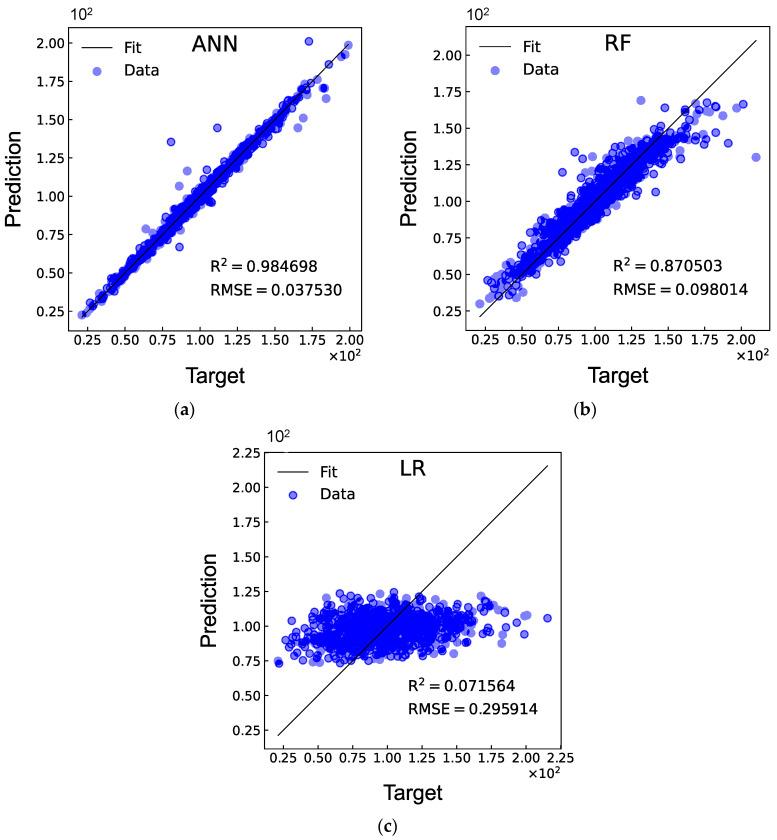
Comparisons of R^2^ and RMSE between the (**a**) ANN, (**b**) RF, and (**c**) LR models for DD laminates with a combined-shape cutout.

This section presents the optimisation of DD laminates with a combined-shape cutout, for which the design variables include the two ply angles ϕ and ψ, the length w and width v of the rectangle, the radius r, and the rotation angle θ. Correspondingly, a total of six inputs are considered for the ANN model. 5000 FEA are conducted to generate the database, and the overall computational time is around 52.64 h. The architecture of the ANN model is established as 6-70-70-70-70-70-70-1 (six hidden layers, each containing 70 neurons). The 10-fold cross-validation results of the ANN models are outlined in Table 3. It can be observed from Figure 12 that the ANN model exhibits the best prediction capability with the values of R^2^ and RMSE equal to 0.985 and 0.038, respectively. Comparisons of the RMSE values between the three types of cutouts illustrate a smooth increase with the increase of the input features. Hence, the prediction accuracy of the ANN models decreases slightly as more features of the cutouts are included.

**Figure 13:**
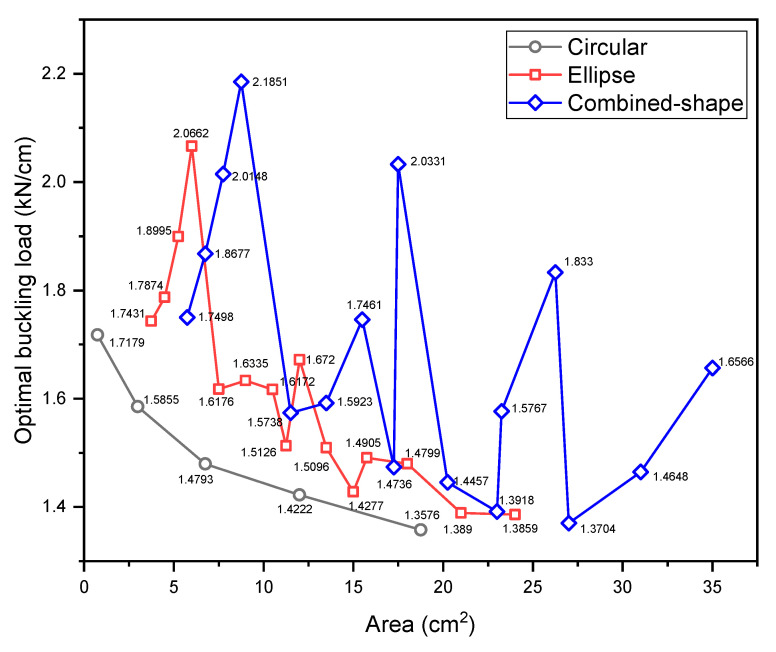
The maximum buckling loads for different types of cutouts.

For the GA optimisation in this case, the population size is 100, the crossover probability is 0.9, and the mutation probability is 0.1. The maximum buckling load converges to 2.180 kN/cm, the optimal ply angles ϕ and ψ are 81° and 35°, respectively, the rotation angle θ is 93°, and the dimension parameters w, v, and r are 10 mm, 80 mm, and 5 mm, respectively. The buckling load of the optimised DD laminate obtained by FEA is 2.185 kN/cm, as shown in Figure 9c. For combined-shape cutouts with predefined sizes, the optimal ply angles ϕ and ψ, and rotation angle θ are also obtained and presented in Table 4. It can be observed that, compared to the elliptical cutout, the utilisation of the combined-shape cutout leads to more noticeable changes in the optimal ply angles with an increase in the cutout size. The maximum buckling loads for the laminates with predefined cutout sizes are shown in Figure 10c. Figure 13 presents the obtained maximum buckling loads for three types of cutouts. As can be seen despite the predefined dimension parameters of the combined-shape cutout, perforated DD laminates with a combined-shape cutout generally exhibit higher buckling resistance compared to those with a circular cutout of the same area, suggesting the potential advantage of using a combined-shape cutout for DD laminates. 

For the i5-CPU Intel processor at 2.5 GHz used in this study, the time required for buckling analyses using FEA and ANN for the three cases is approximately 40 s and 6 × 10^−4^ s, respectively. Consequently, the solution time using ANN is only about 0.0015% of that required by FEA, resulting in a substantial time saving for the GA optimisation process, during which a substantial number of buckling analyses are needed.

## 4. Conclusions

In this paper, the effects of cutout size and shape on the buckling performance of perforated DD laminates are investigated based on an efficient optimisation method. Firstly, the application of machine learning models with two ply angles and cutout parameters as inputs for predicting the buckling resistance of DD laminates is investigated. The combination of the inputs is determined by using a LHS method to ensure comprehensive coverage of the design space. Due to the nature of DD laminates, the machine learning models are better suited for DD laminates compared to conventional QUAD laminates. Comparisons between the developed ANN, RF, and LR models show that the ANN models achieve the highest value of R^2^ and the lowest value of RMSE. The superiority of using ANN models is more obvious when more cutout features are considered. The developed ANNs are then combined with GAs to obtain the maximum buckling loads for the three types of perforated DD laminates. Compared to FEA, the use of the ANN models significantly reduces the computational cost during this optimisation process where a large number of buckling evaluations are performed. Based on the proposed method, the maximum buckling resistance of the perforated DD laminates with varying cutout sizes is studied, considering the three types of cutouts shapes. The obtained results demonstrate that the buckling performance of perforated DD laminates is significantly affected by the cutout size. Increasing the cutout size leads to negligible changes in the optimal values of ϕ and ψ for the DD laminates with circular cutout, but it results in considerable changes in the optimal ply angles when elliptical and combined-shape cutout are used. Furthermore, the obtained results indicate that the application of the combined-shape cutout generally leads to higher buckling resistance in comparison to conventional cutout shapes, providing a promising solution for perforated DD laminates. Although this paper has demonstrated the excellent performance of the proposed method for predicting and optimising the buckling behaviour of perforated DD laminates, its applicability to other structural behaviours, such as fatigue and impact, remains unclear; hence, these areas are proposed as directions for future research.

## Figures and Tables

**Table 1:** Ranges of design variables as input.

Design Variables	Lower Bound	Upper Bound
ϕ	0°	90°
ψ	0°	90°
*d*	10 mm	80 mm
*a*	10 mm	40 mm
*b*	50 mm	80 mm
*θ*	1°	180°
*w*	10 mm	40 mm
*v*	50 mm	80 mm
*r*	1 mm	10 mm

**Table 2:** Material properties.

E_11_	128 GPa
E_22_	11 GPa
G_12_	4.48 GPa
G_13_	4.48 GPa
G_23_	1.53 GPa
*v*_12_	0.25
Ply thickness	0.125 mm

**Table 3:** K-fold cross-validation results of ANN models.

Model	Fold	R^2^ (Train)	RMSE (Train)	R^2^ (Validation)	RMSE (Validation)
Circular cutout	1	0.9987	0.0097	0.9996	0.0099
	2	0.9991	0.0078	0.9996	0.0082
	3	0.9991	0.0075	0.9996	0.0081
	4	0.9990	0.0083	0.9996	0.0091
	5	0.9990	0.0080	0.9996	0.0087
	6	0.9992	0.0061	0.9996	0.0070
	7	0.9993	0.0059	0.9997	0.0066
	8	0.9990	0.0086	0.9995	0.0086
	9	0.9993	0.0069	0.9997	0.0068
	10	0.9996	0.0050	0.9998	0.0055
Elliptical cutout	1	0.9975	0.0185	0.9956	0.0198
	2	0.9987	0.0132	0.9972	0.0158
	3	0.9976	0.0125	0.9957	0.0146
	4	0.9973	0.0103	0.9948	0.0133
	5	0.9985	0.0129	0.9966	0.0173
	6	0.9966	0.0137	0.9949	0.0183
	7	0.9984	0.0130	0.9974	0.0141
	8	0.9974	0.0121	0.9954	0.0141
	9	0.9977	0.0135	0.9972	0.0166
	10	0.9982	0.0138	0.9962	0.0168
Combined-shape cutout	1	0.9989	0.0221	0.9761	0.0493
	2	0.9905	0.0144	0.9794	0.0316
	3	0.9997	0.0099	0.9428	0.0660
	4	0.9986	0.0134	0.9764	0.0432
	5	0.9992	0.0138	0.9885	0.0319
	6	0.9976	0.0128	0.9882	0.0259
	7	0.9997	0.0143	0.9814	0.0407
	8	0.9985	0.0173	0.9773	0.0335
	9	0.9995	0.0079	0.9886	0.0305
	10	0.9982	0.0324	0.9862	0.0470

**Table 4:** Optimum results of the DD laminates with three types of cutouts.

Shape	Area	*Φ*	*Ψ*	*θ*	ANN	FEA
circular	0.75	42	43	−	1.713969	1.7179
	3	43	42	−	1.58486	1.5856
	6.75	39	42	−	1.492208	1.4793
	12	41	41	−	1.420994	1.4222
	18.75	42	40	−	1.350359	1.3576
	27	40	39	−	1.241061	1.2472
	36.75	40	39	−	1.096833	1.0911
	48	40	39	−	0.934522	0.93165
Elliptical	3.75	39	45	83	1.767447	1.7431
	4.5	47	44	77	1.834694	1.7874
	5.25	36	78	82	1.917714	1.8995
	6	37	84	86	2.068347	2.0662
	7.5	39	43	87	1.629106	1.6176
	9	38	43	86	1.658895	1.6335
	10.5	37	43	101	1.625025	1.6172
	11.25	39	42	89	1.520954	1.5126
	12	83	32	91	1.669438	1.672
	13.5	37	43	91	1.511953	1.5096
	15	39	42	89	1.450734	1.4277
	15.75	37	43	113	1.495047	1.4905
	18	36	41	123	1.461465	1.4799
	21	37	44	119	1.405573	1.389
	24	38	38	124	1.368577	1.3859
combined-shape	5.75	36	65	89	1.81204	1.7498
	6.75	37	80	90	1.938178	1.8677
	7.75	90	29	91	2.06614	2.0148
	8.75	81	35	93	2.179764	2.1851
	11.5	36	52	90	1.610907	1.5738
	13.5	34	74	91	1.645834	1.5923
	15.5	83	37	90	1.787188	1.7461
	17.25	43	35	100	1.498023	1.4736
	17.5	29	90	95	2.040842	2.0331
	20.25	67	40	89	1.488566	1.4457
	23	37	45	92	1.387236	1.3918
	23.25	78	39	90	1.625557	1.5767
	26.25	86	38	93	1.808839	1.833
	27	41	46	94	1.39312	1.3704
	31	74	45	90	1.50436	1.4648
	35	81	43	93	1.640859	1.6566

## Data Availability

Data will be made available on request.
